# Supplementary material for: Overrepresentation of New Workers in Jobs with Multiple Carcinogen Exposures in Canada
Source: Int J Environ Res Public Health. 2024 Aug 1;21(8):1013. doi: 10.3390/ijerph21081013 (PMC11354117; doi:10.3390/ijerph21081013)
Supplement: Supplementary file 1 [file ijerph-21-01013-s001.zip › ijerph-3098568-supplementary.pdf]

Supplementary materials

Table S1. Logistic regression results of industries and occupations models.

	Occupation	Industry
**Variables**	**OR[1] 95% CI[1]**	**OR[1] 95% CI[1]**
**Age group (years)**		
25-39	Ref —	Ref —
< 25	3.24 (3.18, 3.31)	3.39 (3.32, 3.46)
40-59	0.54 (0.53, 0.55)	0.55 (0.54, 0.56)
60+	0.46 (0.44, 0.48)	0.48 (0.46, 0.50)
**Immigration status**		
Citizen	Ref —	Ref —
Former immigrant (>10 years)	0.97 (0.94, 1.00)	0.95 (0.92, 0.98)
Recent immigrant (< 10 years)	1.36 (1.32, 1.41)	1.33 (1.29, 1.37)
**Season**		
Spring	Ref —	Ref —
Summer	1.45 (1.41, 1.48)	1.45 (1.41, 1.48)
Fall	1.29 (1.26, 1.33)	1.29 (1.26, 1.32)
Winter	1.10 (1.07, 1.13)	1.10 (1.07, 1.13)
**Occupation**		
Management	Ref —	
Manufacturing and Utilities	2.02 (1.89, 2.16)	
Art, Culture, Rec and Sport	2.58 (2.39, 2.78)	
Business, Finance, and Administration	1.79 (1.69, 1.90)	
Education, Law, Social and Government	1.54 (1.45, 1.63)	
Health	1.10 (1.03, 1.17)	
Natural Resources and Agriculture	3.99 (3.73, 4.27)	
Natural and Applied Science	1.62 (1.52, 1.73)	
Sales and Service	2.34 (2.21, 2.48)	
Trades, Transport and Equipment Operators	2.78 (2.63, 2.95)	
**Industry**		
Public Administration		Ref —
Accommodation and Food Services		1.80 (1.72, 1.89)
Agriculture, Forestry, Fishing, and Hunting		2.51 (2.34, 2.68)
Business, and Building Services		2.60 (2.45, 2.75)
Construction		2.43 (2.32, 2.56)
Education		1.09 (1.03, 1.15)
Finance and Insurance		0.91 (0.85, 0.98)
Health Care and Social Assistance		0.98 (0.93, 1.03)
Information, Culture and Recreation		1.78 (1.68, 1.88)
Manufacturing		1.33 (1.27, 1.40)
Mining, Oil and Gas		1.64 (1.53, 1.75)
Other services		1.71 (1.61, 1.81)

Professional, Scientific and Technical	1.51 (1.43, 1.60
Real estate and rental	1.79 (1.64, 1.96)
Retail trade	1.46 (1.39, 1.53)
Transportation and warehousing	1.61 (1.52, 1.70)
Utilities	0.75 (0.65, 0.85)
Wholesale trade	1.28 (1.20, 1.37)

1OR = Adjusted Odds Ratio, CI = Confidence Interval

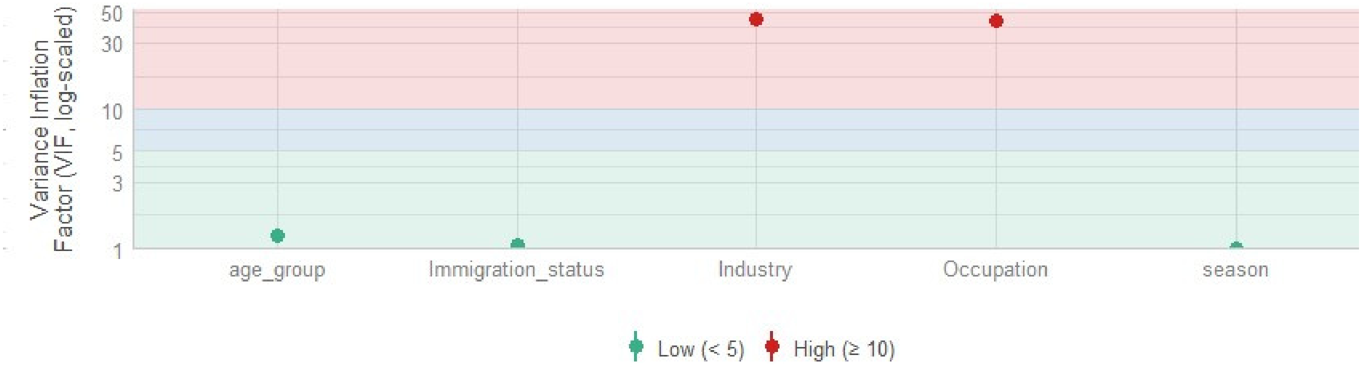

Figure S1. Collinearity plot of the select model variables.
